# Type 2 Diabetes Mellitus Increases the Risk to Hip Fracture in Postmenopausal Osteoporosis by Deteriorating the Trabecular Bone Microarchitecture and Bone Mass

**DOI:** 10.1155/2019/3876957

**Published:** 2019-11-07

**Authors:** Sahar Mohsin, Suneesh Kaimala, Jens Jolly Sunny, Ernest Adeghate, Eric Mensah Brown

**Affiliations:** Department of Anatomy, College of Medicine and Health Sciences, United Arab Emirates University, Al Ain, PO Box 17666, UAE

## Abstract

T2DM is linked to an increase in the fracture rate as compared to the nondiabetic population even with normal or raised bone mineral density (BMD). Hence, bone quality plays an important role in the pathogenesis of skeletal fragility due to T2DM. This study analyzed the changes in the trabecular bone microstructure due to T2DM at various time points in ovariectomized and nonovariectomized rats. Animals were divided into four groups: (I) control (sham), (II) diabetic (sham), (III) ovariectomized, and (IV) ovariectomized with diabetes. The trabecular microarchitecture of the femoral head was characterized using a micro-CT. The differences between the groups were analyzed at 8, 10, and 14 weeks of the onset of T2DM using a two-way analysis of variance and by post hoc multiple comparisons. The diabetic group with and without ovariectomies demonstrated a significant increase in trabecular separation and a decrease in bone volume fraction, trabecular number, and thickness. BMD decreased in ovariectomized diabetic animals at 14 weeks of the onset of T2DM. No significant change was found in connectivity density and degree of anisotropy among groups. The structural model index suggested a change towards a weaker rod-like microstructure in diabetic animals. The data obtained suggested that T2DM affects the trabecular structure within a rat's femoral heads negatively and changes are most significant at a longer duration of T2DM, increasing the risk to hip fractures.

## 1. Introduction

Diabetes mellitus (DM) although identified more than half a century ago as being associated with bone frailty has come to the forefront only within the last decade as an important osteoporosis risk factor. Diabetics suffering from both types are at an increased risk of osteoporosis and fractures [1, 2]. Despite this increased fracture risk, bone fragility remains an underappreciated complication of DM.

Postmenopausal osteoporosis, due to estrogen deficiency, is the most common type of primary osteoporosis in women. Estrogen deficiency imbalances the bone remodeling cycle resulting in increased bone resorption and 25-30% destruction in bone mass during a 5 to 10-year period [3].

In the United States, 10 million individuals > 50 years of age are estimated to have osteoporosis of the hip (based on a *T*-score of ≤−2.5), with about 1.5 million osteoporotic fractures each year [3]. The socioeconomic costs of osteoporosis and associated fractures are high and are expected to increase remarkably over the next decades due to increasing life expectancy. Osteoporotic hip fractures are associated with increased mortality and reduced quality of life and more problematic in patients with diabetes due to compromised bone fracture healing [4]. A recent report has suggested that anxiety negatively affects the HRQoL in patients with diabetes of long duration [5].

The standard diagnostic technique for assessing osteoporosis and monitoring therapy is dual X-ray absorptiometry (DXA) measuring bone mineral density (BMD). BMD can predict femoral bone strength and fracture risk to some extent, but BMD values of patients with and without femur fractures overlap [4, 6]. Osteoporotic changes and increased fractures reported in type 1 DM (T1DM) are linked to a decrease in bone mineral density [2]. However, data on skeletal abnormalities in type 2 DM (T2DM) appear contradictory and the exact explanation to this is still unknown. Individuals with T2DM have low, normal, or increased bone mineral density and yet associated with increased fractures at various skeletal sites [7–9]. Hence, the bone quantity measured as bone mineral density is not the only factor that contributes to osteoporosis, but changes in bone quality should also be considered while predicting fracture risk.

Bone quality involves the analysis of the bone microstructure. In a high-quality bone, the trabeculae are well-connected, greater in number, thicker, and more plate-like [10]. There are less fatigue damage, a higher level of mineralization, and balanced porosity in cortical bones, and the health of osteocytes plays a great role [11]. Osteoporosis deteriorates the microstructure of the bone particularly at trabecular sites, especially in the vertebrae, ribs, and hips.

We hypothesize here that diabetes mellitus is a chronic disease which may also reduce bone quality over a period of time. The aim of the current study is to investigate the effect of T2DM on postmenopausal osteoporosis. Data available for the effect of T2DM on bones is variable, and there is not much data available for combined effects of estrogen deficiency and diabetes.

As the increased risk of fractures in diabetes has a distinct propensity for the proximal end of the femur, the study has evaluated microstructural changes at the femoral head that occurred due to T2DM and lack of estrogen (postmenopausal) and due to the combined effect of T2DM and postmenopausal osteoporosis at various time points. Ovariectomized (OVX) rats were used as a model for postmenopausal osteoporosis.

Microcomputed tomography (micro-CT) is used to investigate changes of the trabecular bone at the femoral head. Micro-CT is a noninvasive technique and regarded as a valuable technique for investigating the microarchitecture of the bone.

## 2. Materials and Methods

### 2.1. Animal Model

Three-month-old female Wistar rats (*n* = 96) were obtained from the animal house facility at United Arab Emirates University for this study. The animals were singly housed in cages under the standard conditions with a 12 h alternating light and dark cycle (22–24°C), in 50–60% humidity and provided with free access to standard rat chow and water ad libitum during the two weeks of acclimatization. All efforts were made to minimize animal suffering and to reduce the number of animals used. All animal procedures were approved by the animal ethical committee at Unite Arab Emirates University (ERA_2017_5597).

### 2.2. Experimental Design

The experimental animals were randomly divided into four groups (*n* = 8 for each group): Gp. I—control/sham-operated, Gp. II—sham-T2DM, Gp. III—OVX, and Gp. IV—T2DM+OVX.

### 2.3. Establishment of Type 2 Diabetic Rat Model

Animals (*n* = 48) were fed a high-calorie diet (D12492 diet; Research Diets, Inc., USA) for 3 weeks followed by the injection of two lower doses of streptozotocin (STZ) (30 mg/kg intraperitoneally) which was administered at weekly intervals. [12]. Three days after the last injection, tail vein blood glucose was measured after fasting for 5 h using a blood glucose meter (Accu-Chek Performa; Roche Diagnostics, USA). Rats having blood glucose > 15 mmol/liter were considered diabetic and were used for our study [12]. Insulin resistance in diabetic animals was further confirmed through an insulin tolerance test [12]. The high-fat diet was continued throughout the experimental period in the diabetic group of animals. The blood glucose concentrations and body weight were monitored fortnightly.

### 2.4. Surgery

Rats underwent bilateral ovariectomies two weeks after the onset of diabetes to make them sex hormone deficient and to stimulate the accelerated loss of bone. The procedure was carried out as per standard protocol [13]. Briefly, the operation was made after placing an anesthetized animal on its dorsal surface. The area of surgery was cleaned with ethanol (Merck, India). A small transverse peritoneal incision was made with a surgical scalpel blade on the middle part of the abdomen slightly towards the right, just near to the second right nipple of the rat to open the peritoneal cavity of the rat. The adipose tissue was pulled away until the right uterine tube and the ovary surrounded by a variable amount of fat were identified. The procedure was repeated for the left ovary through the same incision. After identifying the ovaries and uterine horns, the distal uterine horns were ligated and the ovaries were removed. The distal uterine horns were returned to the peritoneal cavity after removal of ovaries, and the skin was stitched back. The control rats underwent sham procedures only. A postoperative follow-up was carried out on all animals.

All animals (*n* = 96) were sacrificed at 6, 8, and 12 weeks (*n* = 32 for each time interval) after surgery to see the effect of 8, 10, and 14 weeks of the onset of diabetes on bone samples. All bones were dissected out, cleaned, fixed, and kept in the storage for further analysis. Right femurs were dissected out for this study.

### 2.5. Micro-CT Measurements

Femurs were first fixed in buffered formalin, then stored in phosphate-buffered saline (PBS) to be examined by cabinet cone-beam micro-CT (*μ*CT 50, SCANCO Medical AG, Brüttisellen, Switzerland). The test was carried out with a cone beam originating from a 5 *μ*m focal-spot X-ray tube set at a voltage of 70 kVp with a current intensity of 200 *μ*A. The standard 0.5 mm thick aluminum filter was used. The scanned region was approx. 10 mm, and the field of view was 10.24 mm. The integration time was set to 800 ms. The photons were detected by a CCD-based area detector, and the projection data was computer-reconstructed into an image matrix using Scanco 3D-viewer V4.2. For each scan, a stack of 2000 cross-sections was reconstructed. The reconstructed images were 2048 × 2048 pixels each.

### 2.6. Evaluation of Trabecular Structure within the Femoral Head

The samples were segmented based on their grey-scale values in the CT slices. An algorithm was developed to generate a cubical mask in the center of the femoral heads. A volume of interest containing only trabecular bone was extracted for morphometric analysis. The trabecular thickness was computed from the segmented sample using the maximum fitted sphere methods [14, 15]. The pore diameters were then computed applying the same method on the inversed segmented image. Please note that this is not the true pore size but the local thickness of the pores. The script for performing the analysis was started with a one-click operation in the SCANCO evaluation program.

The following structural parameters were measured for the morphometric analysis of trabecular bone: TV—total volume (mm^3^); BV—bone volume (mm^3^); BV/TV—relative bone volume (%); bone surface density which is measured as the ratio of the segmented bone surface to the total volume of the region of interest BS/TV; Conn-Dens—connectivity density, normed by TV (1/mm^3^); SMI—structure model index (0 for parallel plates, 3 for cylindrical rods); Tb.N—trabecular number (1/mm); Tb.Th—trabecular thickness (mm); Tb.Sp: trabecular separation = marrow thickness (mm). These indices are calculated without assuming anything about the shape of the bone (i.e., without plate model assumption). All parameters were calculated three dimensionally (3D) based on counting voxels. Bone mineral density was calculated as mean 1 includes voxel values of everything within the volume of interest (mixture of bone and background) scan which were calibrated for bone in units of mg HA/ccm.

### 2.7. Statistical Analysis

Data were analyzed by two-way analysis of variance and unpaired *t*-tests using GraphPad Prism 5 (GraphPad Software Inc., San Diego, CA, USA). Bonferroni's multiple comparison test was used to assess the difference between the groups and also if there is any change in defined trabecular structural parameters over different time periods. Data are expressed as the means ± SD. Statistical significance was established at *p* < 0.05. Adjusted *p* value (^∗^*p* < 0.05, ^∗∗^*p* < 0.01).

## 3. Results

The blood glucose levels (mean ± SD) for diabetic rats range between 21.30 ± 8.16 to 26.50 ± 1.02 (mmol/liter) and 19.98 ± 8.05 to 24.63 ± 6.58 (mmol/liter) for Gp. II and Gp. IV, respectively. The blood glucose level for nondiabetic rats in Gp. I was 6.3 ± 0.26 to 6.62 ± 0.53 (mmol/liter) and 6.15 ± 2.03 to 6.49 ± 0.88 (mmol/liter) in Gp. III (see complete data in the supplementary file ([Supplementary-material supplementary-material-1])).

**Figure 1:**
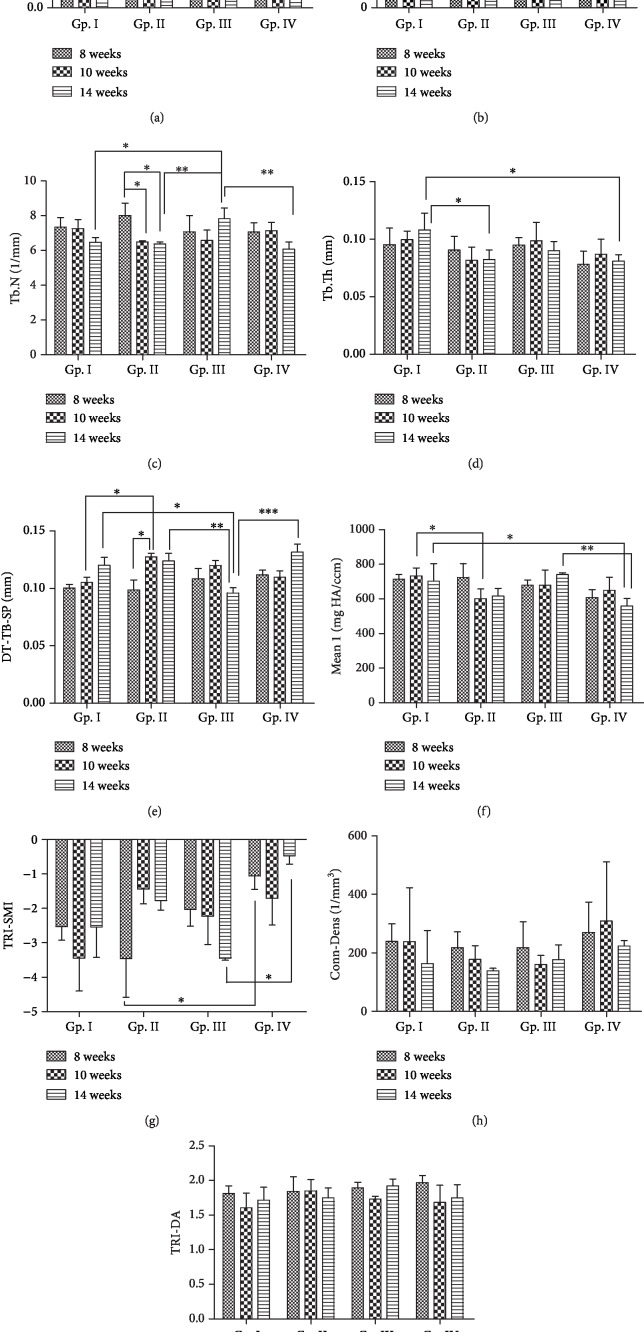
Plots of changes in various structural parameters of trabecular bone: (a) bone volume/total volume BV/TV, (b) bone surface density (BS/TV), (c) trabecular number (Tb.N), (d) trabecular thickness (Tb.Th), (e) trabecular separation (Tb.Sp), (f) mean 1 (vBMD), (g) structural model index (SMI), (h) connectivity density (Conn-Dens), and (i) degree of anisotropy (DA) of a rat's femur head from Gp. I—control/sham-operated, Gp. II—sham-T2DM, Gp. III—OVX, and Gp. IV—T2DM+OVX which were compared over the period of 8, 10, and 14 weeks of onset of diabetes. Two-way ANOVA was performed with Bonferroni's posttest multiple comparison test using GraphPad Prism 5. Adjusted *p* value (^∗^*p* < 0.05, ^∗∗^*p* < 0.01). Error bars = mean ± SD.

**Figure 2:**
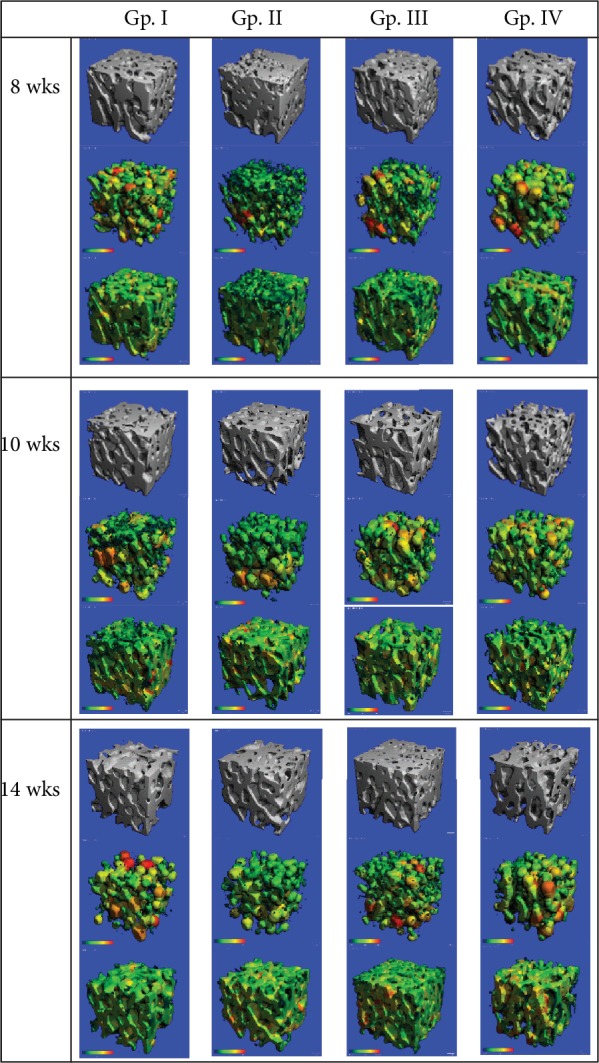
Representation of 3D microarchitecture of the trabecular bone at the head of the femur from 12 rats from four groups: Gp. I—control/sham-operated, Gp. II—sham-T2DM, Gp. III—OVX, and Gp. IV—T2DM+OVX obtained by micro-CT examination at 6, 8, and 10 weeks after surgery.

Graphs for all the measured structural parameters and 3D images of the micro-CT scans of the trabecular bone from each of the four groups at three different time points are shown in Figures 1 and 2, respectively.  Table 1 shows the mean ± SD values and percentage differences for the significant data for all the measured structural parameters. 3D evaluation of trabecular structure within a rat's femur head was compared over the period of 8, 10, and 14 weeks of the onset of T2DM in Gp. I—control/sham-operated, Gp. II—sham-T2DM, Gp. III—OVX, and Gp. IV—T2DM+OVX. The total number of animals in each group was 8 for any one-time period.

The BV/TV decreased *p* < 0.05 in Gp. II and Gp. IV bone samples when compared with controls in Gp. I after 10 and 14 weeks of the onset of diabetes, respectively. The BV/TV in Gp. II (14 wks) sham-diabetic animals was significantly less (*p* < 0.05) than nondiabetic ovariectomized animals from Gp. III (14 wks). A statistically significant decrease in BV/TV was also observed in Gp. IV when compared with Gp. III (^∗∗^*p* < 0.01) at 14 weeks of the onset of DM (Figure 1(a)).

The BS/TV increased in Gp. III at 14 weeks of the onset of diabetes which was found when compared with respective control in GP. I (*p* < 0.05) (Figure 1(b)).

The Tb.N decreased (*p* < 0.05) in Gp. II at 10 and 14 weeks of the onset of DM when compared with the dataset obtained from 8-month diabetic animals from the same group. The Tb.N was found to decrease (*p* < 0.01) in Gp. IV after 14 weeks of exposure to diabetes as compared to its respective control in Gp. III. However, the Tb. N in the 14-week bone samples obtained from Gp. III was significantly higher (*p* < 0.05) when compared with those from Gp. I and Gp. II and with those obtained from Gp. IV (*p* < 0.01) (Figure 1(c)). The Tb.Th (Figure 1(d)) in Gp. II and Gp. IV at 14 weeks was significantly lower (*p* < 0.05) than control in Gp. I.

The porosity within the trabecular bone was measured as Tb.Sp. It increased (*p* < 0.05) in Gp. II at 10 weeks when compared with its respective control in Gp. I and also when compared with that in Gp. III at 14 weeks (*p* < 0.01). Additionally, increased trabecular separation was found in the diabetic group (Gp. II) with an increase in the duration of diabetes and the change was significant (*p* < 0.05) between 8 and 10 weeks of the onset of diabetes in Gp. II. Trabecular separation in Gp. IV was significantly higher (*p* < 0.001) than that in Gp. III at 14 weeks of the onset of diabetes (Figure 1(e)). The data for Gp. III was heterogeneous, and though the trabecular separation increased with time (8-10 wks), the change was not significant, and at 14 weeks, the bone samples showed a decrease in the trabecular separation when compared with controls (Gp. I) (*p* < 0.05 at the same time point).

Mean 1 which represents vBMD decreased significantly in 14-week bone samples from Gp. IV when compared with those from Gp. I and Gp. III with *p* < 0.05 and *p* < 0.01, respectively (Figure 1(f)).

Higher negative values for SMI were found in the nondiabetic control groups—Gp. I and Gp. III. The SMI was found to increase in Gp. IV after 8 and 14 weeks of the onset of diabetes when compared with those in Gp. II and Gp. III at the same time points (Figure 1(g)).

No significant difference has been found in any comparison for connectivity density (Figure 1(h)) and the degree of anisotropy (DA) (Figure 1(i)). The interaction between different groups and the effect of the duration of time on different groups were also considered to be nonsignificant (*p* > 0.05). No statistically significant change in any of the structural parameters was found with time alone in control (Gp. I).

## 4. Discussion

Fragility fractures are a common complication of osteoporosis affecting the elderly population predominantly women after estrogen loss in postmenopausal age. The bone loss associated with estrogen deficiency is generally attributed to an imbalance between bone resorption and formation results in the loss of bone mass and deterioration of trabecular bone microarchitecture [16–18].

The importance of PMO is very clear as with the increase in the aging population, the complications such as the hip fractures will treble to over six million a year by 2050 [19]. T2DM is more common with advancing age and, therefore, frequently coexists with age-related bone loss [20, 21]. Diabetes has an increased prevalence of risk factors for falls and subsequent injuries, including poor vision, peripheral neuropathy, and stroke. Data has shown that an increased tendency of falls and a higher risk of injury following a fall do not fully account for the greater risk of fracture in diabetes and people suffering from T2DM are at an increased risk of fragility fractures despite normal or increased bone mineral density. Hence, recent research is focused on analyzing changes in the bone microarchitecture that deteriorates bone quality and could be an important factor contributing to diabetic osteopathy.

Patients suffering from diabetes have an increased incidence of fragility fractures at an early age as compared to the nondiabetic population [21–23]. The estimated risk ratio for diabetes and hip fracture is 1.38 (95% CI, 1.25–1.53) for T2DM [22]. Trabecular bone loss is more prominent in PMO due to its large surface to volume ratio, and it shows a higher turnover rate than the cortical bone [18, 24, 25]. This study characterized using micro-CT the changes in the trabecular architecture within the head of a rat's femur, due to different duration of T2DM, and investigated the effect of T2DM on postmenopausal osteoporosis. The data in this study were collected by using an animal model; however, further human studies will be necessary to confirm these results.

The ovariectomized rat model used in this study is a well-established animal model of postmenopausal osteoporosis because the bone loss in these animals is considered to mimic that of postmenopausal women [24]. The study examined the changes in the trabecular bone microarchitecture within the head of the femur from control/sham-operated, sham-T2DM, OVX, and T2DM+OVX female mature rats using micro-CT. Micro-CT examination is a noninvasive, nondestructive way of examining the microarchitecture of the bone at high resolution [26, 27].

Bone structural parameters measured were similar to histomorphometry analysis, such as the bone volume fraction (BV/TV), bone surface density (BS/TV), trabecular number (Tb.N), trabecular thickness (Tb.Th), and trabecular separation (Tb.Sp). Nonmetric parameters such as the structural model index (SMI), connectivity density (Conn-Dens), degree of anisotropy (DA), mean 1, or vBMD were also measured. [27].

The BV/TV indicates the fraction of a given volume of interest (VOI, i.e., the total volume (TV)) that is occupied by the mineralized bone (bone volume). It was evaluated to detect relative changes if any in bone volume density that occurred following ovariectomy (OVX) and diabetes (T2DM). The results have shown that T2DM negatively affects bone volume density as it is significantly decreased in bones of diabetic animals with and without ovariectomy. The bone volume density significantly decreased with an increase in the duration of diabetes with *p* < 0.01 at 14 weeks of the onset of DM in ovariectomized animals with diabetes (Table 1).

The bone surface density (BS/TV) which is measured as a ratio of the segmented bone surface to the total volume of the region of interest [14, 15] was increased in ovariectomized rats without diabetes (Gp. III) after 14 weeks of exposure to diabetes compared to its respective control from Gp. I in this study. The bone surface is affected by the activity of osteoclasts and osteoblasts where resorption lacunae have more perimeter per unit length than osteoid covered or quiescent bone [28]. This represents increased bone turnover in ovariectomized animals as reported by earlier studies [29, 30].

The number of trabeculae (Tb.N) decreased after 14 weeks of the onset of diabetes in the postdiabetic ovariectomized group when compared with their respective ovariectomized nondiabetic controls. Additionally, we found a decrease in the number of trabeculae with an increase in the duration of diabetes in the nonovariectomized diabetic group. Trabecular thickness also decreased under the influence of DM in diabetic rats with or without ovariectomy as found in an earlier study [24].

However, unlike earlier studies [31–33], we found an increase in the trabecular number in samples obtained from an ovariectomized animal. This difference in results could be due to a transient stage of osteoporosis in our samples where trabeculae were not completely resorbed and were in the process of breakdown.

Trabecular separation means an increase in the distance between the adjacent trabeculae and requires perforation and removal of whole trabecular elements. This study is consistent with previous studies which showed that bones are becoming increasingly porous with an increase in trabecular separation in both diabetic groups with and without ovariectomies [34]. The trabecular separation increase was the most significant (*p* < 0.001) in ovariectomized animals at 14 weeks of the onset of diabetes. Trabecular separation increased with an increase in the duration of diabetes in the nonovariectomized group with significant change observed in bones at 10 weeks of the onset of DM as compared to bones at 8 weeks of the onset of DM. [34]. Pritchard et al. [34] also found an increase in the average whole size within the trabecular bone network at the distal radius and suggested it contributes to higher fracture risk in type 2 diabetic population. Kerckhofs et al., [35] on the contrary, found an increase in the thickness of trabeculae and no significant change in trabecular separation in the proximal tibia of diabetic mice when compared to controls.

Mean 1 or vBMD represents what is often called “volumetric bone mineral density,” more precisely called apparent bone mineral density. Specifically, it is the total bone mineral content contained within the volume of interest divided by the total volume (TV) of the region of interest. It is the recommended method [27] for reporting BMD for a cancellous bone as it relates directly to bone strength. It shows trends similar to BV/TV.

Data available on skeletal abnormalities in T2DM and its direct relationship with BMD are contradictory, and the exact explanation of this is still unknown. In different studies, bone mineral density values have increased, decreased, or remained normal [36]. Petit and colleagues [8] reported a higher BMD in elderly patients with T2DM when compared to age-matched non-DM volunteers. In contrast, several other investigators reported a negative effect of T2DM on BMD [9, 37].

This study found that BMD tends to decrease with an increase in the duration of diabetes in nonovariectomized diabetic animals, and significant change (*p* < 0.05) was recorded at 8 weeks of the onset of DM when compared with its respective controls. Most significant (*p* < 0.01) negative changes in BMD were seen in the postovariectomized diabetic group after 14 weeks of the onset of DM when compared with its respective control in nondiabetic ovariectomized control. The results of this study show that T2DM affects the BMD negatively as reported in earlier studies [9, 37, 38].

However, we did not find any significant decrease in BMD in the head of the femur from the ovariectomized nondiabetic group as reported by others [30] for the proximal tibial metaphysis in the ovariectomized rats compared to the control group. Substantial heterogeneity in the data is most likely due to differences in the study design and use of the different animal models or skeletal sites.

The structure model index (SMI) is a parameter defined to describe a “plate-like” and “rod-like” architecture of trabeculae within the cancellous bone. It is calculated by means of three-dimensional image analysis based on a differential analysis of the triangulated bone surface. The relative proportion of rods to plates in the trabecular bone is thought to be important for the bone's mechanical competence, with plates considered to be mechanically superior to rods. The deterioration of the cancellous bone structure due to aging and disease is characterized by conversion from plate elements to rod elements. The most real trabecular structures will lie somewhere between the ideal plate and ideal rod structure, and the value lies between 0 and 3, depending on the volume ratio of rods and plates. It is possible that samples may have similar volume density but varying SMI number depending upon the number of the plate-like and rod-like architecture of trabaculae.

The results of our study show a significant increase in SMI values at the head of the femur obtained from postdiabetic ovariectomized animals when compared to nonovariectomized animals with diabetes and with ovariectomized animals without diabetes at eight and fourteen weeks, respectively. The increase in SMI value suggests that the trabecular architecture is changing from more plate-like to weaker rod-like as trabeculae undergo perforation and/or thinning mechanisms [39].

Higher negative values were found in the nondiabetic control groups Gp. I and Gp. III which correspond to high BV/TV values in these groups as found. Hildebrand et al. [40] also showed that the trabecular structure is predominantly plate-like in the head of the femur. Negative SMI values are also reported in an earlier study [40] in the distal femoral metaphysis with BV/TV greater than 35%. As bone volume fraction decreases, trabeculae tend to become more rod-like. However, at very high or low BV/TV values, the SMI may be outside of the defined range [27]. More negative values represent a more plate-like, stronger lattice and are being associated with the greatest strength and less fracture risk [41].

The connectivity density (Conn-Dens) represents one aspect of how trabeculae contribute to bone strength by estimating their interconnectivity. It represents how many branches between nodes can be cut before the structure is completely separated. The connectivity density tends to decrease with aging in each group in this study; however, no statistically significant difference was found within or in comparison to other groups.

The degree of anisotropy (TRI-DA) is described as 1 being isotropic and >1 being anisotropic. Bone tissue is described to be an anisotropic material which means that it can show different mechanical behaviors to applied load in different directions [42]. All the groups in this study showed an anisotropic bone structure with no significant change due to ovariectomies or due to diabetes in the degree of anisotropy within the head of the femur unlike other studies [2, 43] which showed an increase in the degree of anisotropy with an advanced stage of postmenopausal osteoporosis due to selective bone loss. The difference in the result could be due to the small sample size in each group in this study.

## 5. Conclusions

This study showed a significant effect of diabetes on trabecular bone architecture and on bone mineral density in the head of the femur obtained from mature female Wistar rats with and without ovariectomies. Hence, T2DM should be considered an important risk factor for hip fractures. From a clinical perspective, the elderly female population with T2DM is at higher risk of fracture; therefore, there is a need to correlate the measurement of bone mass with the measurements of structural parameters. Most changes in trabecular microstructure and in bone mineral density were found at the longer duration of diabetes. Better glycaemic control at an earlier stage of diabetes may prevent or delay the deterioration of bone microarchitecture and preserve both bone quality and bone quantity. A better understanding of the bone microstructure and metabolism will help us find various mechanisms of skeletal fragility involved in diabetes and would be helpful in improving its diagnosis, treatment, and assessment of the efficacy of the osteoporosis therapy.

## Figures and Tables

**(a):** 

VOX BV/TV (%)			
	8 weeks	10 weeks	14 weeks
Gp. I	0.60 ± 0.02	0.63 ± 0.05	0.58 ± 0.09
Gp. II	0.61 ± 0.09	0.48 ± 0.06^a^	0.47 ± 0.06^b^
Gp. III	0.57 ± 0.03	0.57 ± 0.08	0.63 ± 0.00
Gp. IV	0.49 ± 0.05	0.53 ± 0.08	0.44 ± 0.04^c,d^

^a^% difference to Gp. I (-23.80) (^∗^*p* < 0.05). ^b^% difference to Gp. I (-24.1) (^∗^*p* < 0.05). ^c^% difference to Gp. III (-25.3) (^∗^*p* < 0.05). ^d^% difference to Gp. III (-30.15) (^∗∗^*p* < 0.01).

**(b):** 

TRI-BS/TV (mm^−1^)			
	8 weeks	10 weeks	14 weeks
Gp. I	14.69 ± 1.02	14.21 ± 1.14	12.88 ± 0.86
Gp. II	14.77 ± 0.74	13.93 ± 0.47	14.07 ± 0.35
Gp. III	14.02 ± 1.30	13.33 ± 0.62	15.00 ± 1.22^a^
Gp. IV	15.14 ± 1.11	14.90 ± 1.91	13.67 ± 0.32

^a^% difference to Gp. I (16.52) (^∗^*p* < 0.05).

**(c):** 

DT-TB.N (1/mm)			
	8 weeks	10 weeks	14 weeks
Gp. I	7.35 ± 0.54	7.25 ± 0.52	6.47 ± 0.27
Gp. II	8.01 ± 0.72	6.48 ± 0.07^a^	6.37 ± 0.11^b^
Gp. III	7.07 ± 0.94	6.57 ± 0.60	7.83 ± 0.61^c,d,e^
Gp. IV	7.06 ± 0.53	7.13 ± 0.49	6.08 ± 0.41

^a^% difference to Gp. I (21.02) (*p* < 0.05). ^b^% difference to 8 weeks in Gp. II (-19.10) (*p* < 0.05). ^c^% difference to 8 weeks in Gp. II (-20.47) (*p* < 0.05). ^d^% difference to Gp. II (22.91) (*p* < 0.01). ^e^% difference to Gp. IV (28.78) (*p* < 0.01).

**(d):** 

DT-Tb.Th (*μ*m)			
	8 weeks	10 weeks	14 weeks
Gp. I	0.09 ± 0.01	0.09 ± 0.00	0.10 ± 0.01
Gp. II	0.09 ± 0.01	0.08 ± 0.01	0.08 ± 0.00^a^
Gp. III	0.09 ± 0.00	0.09 ± 0.01	0.09 ± 0.00
Gp. IV	0.07 ± 0.01	0.08 ± 0.01	0.08 ± 0.00^b^

^a^% difference to Gp. I (-20) (*p* < 0.05). ^b^% difference to Gp. I (-20) (*p* < 0.05).

**(e):** 

DT-Tb.Sp (*μ*m)			
	8 weeks	10 weeks	14 weeks
Gp. I	0.10 ± 0.00	0.10 ± 0.00	0.12 ± 0.01
Gp. II	0.09 ± 0.01	0.12 ± 0.00^a,b^	0.12 ± 0.01
Gp. III	0.10 ± 0.01	0.11 ± 0.00	0.09 ± 0.00^c,d,e^
Gp. IV	0.11 ± 0.00	0.10 ± 0.00	0.13 ± 0.01

^a^% difference to Gp. I (20) (*p* < 0.05). ^b^% difference to Gp. I (-25) (*p* < 0.05). ^c^% difference to 8 weeks in Gp. II (20) (*p* < 0.05). ^d^% difference to Gp. II (33) (*p* < 0.01). ^e^% difference to Gp. IV (44) (*p* < 0.001).

**(f):** 

Mean 1/v BMD			
	8 weeks	10 weeks	14 weeks
Gp. I	713.91 ± 28.29	733.09 ± 45.67	703.05 ± 100.41
Gp. II	723.56 ± 80.66	601.41 ± 57.03^a^	615.87 ± 45.78
Gp. III	679.41 ± 30.16	678.59 ± 87.01	740.24 ± 10.18
Gp. IV	607.56 ± 45.38	649.28 ± 76.17	558.88 ± 43.70^b,c^

^a^% difference to Gp. I (-17.97) (*p* < 0.05). ^b^% difference to Gp. I (-20.5) (*p* < 0.05). ^c^% difference to Gp. III (-24.5) (*p* < 0.01).

**(g):** 

TRI-SMI			
	8 weeks	10 weeks	14 weeks
Gp. I	‐2.52 ± 0.67	‐3.44 ± 1.64	‐2.53 ± 1.52
Gp. II	‐3.45 ± 1.95_a_	‐1.42 ± 0.75	‐1.77 ± 0.48
Gp. III	‐2.02 ± 0.85	‐2.22 ± 1.43	‐3.44 ± 0.10^b^
Gp. IV	‐1.05 ± 0.68	‐1.70 ± 1.35	‐0.46 ± 0.42

^a^% difference to Gp. IV (-69) (*p* < 0.05). ^b^% difference to Gp. IV (-86) (*p* < 0.05).

**(h):** 

Conn-Dens			
	8 weeks	10 weeks	14 weeks
Gp. I	239.30 ± 60.24	238.45 ± 183.92	163.451 ± 112.91
Gp. II	217.70 ± 54.22	177.56 ± 47.33	138.773 ± 8.98
Gp. III	217.53 ± 88.57	160.22 ± 31.02	176.888 ± 50.66
Gp. IV	270.09 ± 103.62	308.704 ± 201.93	223.662 ± 18.52

## Data Availability

The data used to support the findings of this study are available from the corresponding author upon request.

## Supplementary Materials

Supplementary Materials Supplementary data shows the mean ± SD of body weight (gms) and blood glucose level (mmol/l) changes in GP. I, GP. II, GP. III, and GP. IV at 8, 10, and 14 weeks of the onset of diabetes before sacrifice.
