# Supplementary material for: Type 2 Diabetes Mellitus Increases the Risk to Hip Fracture in Postmenopausal Osteoporosis by Deteriorating the Trabecular Bone Microarchitecture and Bone Mass
Source: J Diabetes Res. 2019 Nov 7;2019:3876957. doi: 10.1155/2019/3876957 (PMC6878775; doi:10.1155/2019/3876957)
Supplement: Supplementary Materials — Supplementary data shows the mean ± SD of body weight (gms) and blood glucose level (mmol/l) changes in GP. I, GP. II, GP. III, and GP. IV at 8, 10, and 14 weeks of the onset of diabetes before sacrifice. [file 3876957.f1.pdf]

| Groups | 8 (wks) | | 10 (wks) | | 14 (wks) | |
|---|---|---|---|---|---|---|
| | Blood Glucose (Mmol/l) | Body Weight (gm) | Blood Glucose (mmol/l) | Body Weight (gm) | Blood Glucose (Mmol/l) | Body Weight (gm) |
| GP. I | 6.3 ± 0.26 | 251.8 ± 39.7 | 6.34 ± 0.46 | 243.6 ± 26.2 | 6.62 ± 0.53 | 255.5 ± 29.8 |
| GP.II | 21.30 ± 8.16 | 318.8 ± 67.1 | 24.50 ± 2.90 | 203.5 ± 25.98 | 26.50 ± 1.02 | 229.8 ± 48.5 |
| GP.III | 6.15 ± 2.03 | 296.7 ± 13.7 | 6.49 ± 0.88 | 261.3 ± 22.1 | 6.33 ± 0.58 | 252.9 ± 27.4 |
| GP.IV | 24.63 ± 6.58 | 256 ± 40.6 | 19.98 ± 8.05 | 247.3 ± 23.5 | 23.29 ± 4.58 | 262.1 ± 20.0 |

**Supplementary data file**: The table shows the Mean ± S.D of body weight (gms) and blood glucose level (mmol/l) changes in GP. I, II, III and IV at 8, 10 and 14 weeks of the onset of diabetes before sacrifice.
